# Facilitated endospore detection for *Bacillus* spp. through automated algorithm‐based image processing

**DOI:** 10.1002/elsc.202100137

**Published:** 2021-12-10

**Authors:** Riekje Biermann, Laura Niemeyer, Laura Rösner, Christian Ude, Patrick Lindner, Ismet Bice, Sascha Beutel

**Affiliations:** ^1^ Institute of Technical Chemistry Leibniz University Hannover Hannover Germany; ^2^ Institute of Technical Chemistry Biochem Zusatzstoffe Handels‐ und Produktionsgesellschaft mbH Lohne Germany

**Keywords:** automated spore detection, cell counting, digital image processing, DPA assay, spore detection

## Abstract

*Bacillus* spp. endospores are important dormant cell forms and are distributed widely in environmental samples. While these endospores can have important industrial value (e.g. use in animal feed as probiotics), they can also be pathogenic for humans and animals, emphasizing the need for effective endospore detection. Standard spore detection by colony forming units (CFU) is time‐consuming, elaborate and prone to error. Manual spore detection by spore count in cell counting chambers via phase‐contrast microscopy is less time‐consuming. However, it requires a trained person to conduct. Thus, the development of a facilitated spore detection tool is necessary. This work presents two alternative quantification methods: first, a colorimetric assay for detecting the biomarker dipicolinic acid (DPA) adapted to modern needs and applied for *Bacillus* spp. and second, a model‐based automated spore detection algorithm for spore count in phase‐contrast microscopic pictures. This automated spore count tool advances manual spore detection in cell counting chambers, and does not require human overview after sample preparation. In conclusion, this developed model detected various *Bacillus* spp. endospores with a correctness of 85–89%, and allows an automation and time‐saving of *Bacillus* endospore detection. In the laboratory routine, endospore detection and counting was achieved within 5–10 min, compared to up to 48 h with conventional methods. The DPA‐assay on the other hand enabled very accurate spore detection by simple colorimetric measurement and can thus be applied as a reference method.

**Abbreviations**
CFU: colony forming units
DPA: dipicolinic acid

## 1 INTRODUCTION

Bacterial endospores are widely distributed metabolically dormant cell forms that guarantee population maintenance under harsh environmental conditions and withstand stress, such as heat, UV radiation or high pressure [1, 2]. *Bacillus* spp. form endospores under nutrient depletion or environmental stress, involving a complex biochemical process resulting in the lysis of the mother cell and release of an endospore [3, 4]. During endospore maturation, a high content (5‐15% dry mass) of dipicolinic acid (pyridine‐2,6‐dicarboxylic acid, DPA) is formed intracellular [5]. The dicarbonic acid DPA chelates with Ca^2+^ and primarily confers the extreme heat‐resistance of endospores [6, 7]. The matured condensed endospores show a high self‐reflection in phase‐contrast microscopy mainly due to the DPA content [8]. Furthermore, DPA only occurs in spores, making it a well‐suited biomarker for endospore detection [9].

*Bacillus* spp. endospores are used in biotechnological products such as probiotics or other nutritional supplements [10], but can also pose a possible health threat due to pathogenic strains. Therefore, an efficient endospore detection tool in a biotechnological process or as potential contamination control is of great importance. A standardized method for spore detection, often used for quality control, is the determination of colony forming units (CFU). Nonetheless, this approach is prone to errors by dilution, sampling, and interpretation of the results. Moreover, the growth of plated bacteria and their morphology, density and motility lead to an underestimation of CFU and consequently to a decreased bacterial cell count. CFU determination is a time‐consuming and elaborate method for spore detection due to sample preparation and incubation time [11, 12, 13]. Furthermore, endospores can also be marked by staining techniques such as Schaeffer‐Fulton to distinct between vegetative cells and endospores [14, 15]. As mentioned before, DPA acts as a suitable biomarker and is often used for the spore detection as well [9]. DPA can either be detected by a historic colorimetric assay from the 1950‐1960s, using ammonium iron(II) sulfate [16, 17] or in more recent publications with lanthanides such as europium(III) or terbium(III) [18, 19, 20, 21, 22]. Those assays have no special technical requirements and lead to a significant time‐saving compared to CFU. The ammonium iron(II) sulfate assay has the advantage that the used chemicals are inexpensive and can be disposed of easily. Nonetheless, it has sunk into oblivion, maybe due to poor applicability and reproducibility caused by the inept antioxidant ascorbic acid. DPA assays with lanthanides are more expensive and need a separate disposal. Lanthanide assays are better suitable for low DPA concentrations, making it a more sensitive method than the ammonium iron(II) sulfate assay [21].

**PRACTICAL APPLICATION**

The detection of endospores can be time‐consuming and often impedes real‐time detection. So far, the current state‐of‐the‐art method for spore quantification is the elaborate CFU determination. In this work, two methods, namely an historical assay for the dipicolinic acid (DPA) content determination adapted to fit modern requirements and a novel automated counting model are presented, which both shorten the time and workload of *Bacillus* endospore detection. Particularly, the counting model facilitates the spore determination in the laboratory routine with minimal technical effort. Additionally, this counting model could be used as an at‐line automation tool for running bioprocesses or spore production monitoring. In the future, the developed counting model could be coupled with deep learning of artificial intelligence (AI) to further improve its spore detection performance further. The adapted DPA‐assay on the other hand, also showed also high accuracy. Thus, it can generally serve as a suitable fast applicable reference method for spore detection rather than CFU determination.

Other detection methods include Raman spectroscopy [23, 24, 25], fluorimetry [21], laser spectroscopy [26], or flow‐cytometry [27]. Additionally, spores can be detected chromatographically by GC [28] or HPLC [29]. While these methods enable precise endospore detection and quantification, they are often limited by expensive equipment and the requirement for trained staff.

In fact, the most obvious form of endospore detection may be microscopy, specifically after Schaeffer‐Fulton staining or by phase‐contrast microscopy [23, 30]. Microscopic detection of spores is an easy method requiring minimal technical effort. However, this method relies on personnel that manually performs laborious cell counting in a cell counting chamber. To overcome the existing hurdles of conventional endospore detection tools, as explained above, innovative solutions are needed. For example, automated microscopic methods could simplify and speed up spore detection and also minimize the workload and human‐sourced errors [31]. A semi‐automated fungus spore detection method was developed by Li et al. using an algorithm‐based automatic spore counter combined with a human oversight element resulting in a correct spore detection of 90% [32]. For *Bacillus subtilis*, a semi‐automated image processing algorithm was developed for purity control of spore samples. Here, a combination of Schaeffer‐Fulton staining and bright‐field as well as fluorescence microscopy was used for spore detection [33]. Fully automated microscopy spore detection algorithms were reported for fungi by Lei et al. [34], Korsnes et al. [35] and Gao et al. [36] based on microscopy spore detection.

In this work, an image processing model for spore cell detection and counting from phase‐contrast optical micrographs was developed and applied through the development of a fully automated image processing algorithm. This algorithm detects and counts *Bacillus* spp. endospores within 5‐10 min and is thus likely to tremendously expedite and facilitate spore detection in comparison to conventional methods such as CFU determination.

## 2 MATERIALS AND METHODS

### 2.1 Chemicals

All used consumables were obtained by Sarstedt AG & Co. KG (Nümbrecht, Germany), VWR International GmbH (Darmstadt, Germany), Fisher Scientific GmbH (Schwerte, Germany), neoLab Migge (Heidelberg, Germany) and Sartorius AG (Göttingen, Germany). Bulk chemicals were purchased by Carl Roth GmbH & Co. KG (Karlsruhe, Germany), Sigma‐Aldrich (St. Louis, Missouri, USA), Merck (Darmstadt, Germany), AppliChem (Darmstadt, Germany) and Thermofisher Scientific (Schwerte, Germany). For the preparation of purified water, a Sartorius Arium device (Sartorius Stedim Biotech, Göttingen, Germany) was used.

### 2.2 Bacterial strains and culture conditions

For this study, three different *Bacillus* species were used. Two of the *Bacillus* spp. (*B. coagulans*, *B. licheniformis*) were provided by Biochem Zusatzstoffe Handels‐ und Produktionsges. mbH, Lohne, Germany. Previously mentioned species were provided as spray dried powder. The third species (*B. subtilis* DSM 168) was stored as a glycerol culture with 20% v/v glycerol at ‐80°C. Cultivation of the bacteria was conducted in shake flasks with baffles at 30°C (*B. subtilis*), 37°C (*B. licheniformis*) or 50°C (*B. coagulans*) at 150 rpm in standard I medium no. 453 after DSMZ (German Collection of Microorganisms, *Deutsche Sammlung von Mikroorganismen und Zellkulturen*) [medium composition in [g mL^−1^]: peptone (meat) 7.8, peptone (caseine) 7.8, yeast extract 2.8, NaCl 5.6, D(+) glucose 1.0; pH 7.5 in purified water]. The strains present as spray dried powder were activated by suspending and swelling the cell pellet in 500 μL 0.9% w/v sterile saline solution for 30 min. The suspensions then were diluted 1:10 in media and were treated accordingly as described above for the cultivation of the bacteria.

### 2.3 Petroff cell counting chamber

The total cell count and the number of endospores were determined using a Petroff chamber (depth 0.02 mm). If necessary, samples were diluted with 0.9% w/v saline solution. For the cell count at least four counting chambers with each five large squares consisting of 16 small squares were counted. Endospores and vegetative cells were counted using the same sample to generate the total cell count. For the detection of endospores and vegetative cells a phase‐contrast microscope (microscope: Olympus CX41RF and condenser: CX‐PCD) was used with a 40 × magnification. The cell number was calculated as depicted in Equation (1).

(1) $$\begin{array}{ccc} & & \mathrm{cell}\;\mathrm{count}\;[{\mathrm{cells}\;\mathrm{mL}}^{-1}]\\ & & \;=\frac{\emptyset \;\mathrm{cell}\;\mathrm{count}\left(\mathrm{small}\;\mathrm{squares}\right)\times \mathrm{dilution}\;\mathrm{factor}}{\mathrm{volume}\;\mathrm{small}\;\mathrm{square}\left(5\times 10^{-8}\;\mathrm{mL}\right)} \end{array}$$

### 2.4 Colony forming units

The spread plate method was used to determine the CFU. Petri dishes containing PNY‐medium [medium composition in [g mL^−1^]: peptone (caseine) 5.0, yeast extract 5.0, D(+) glucose 5.0, KH_2_PO_4_ 0.5, K_2_HPO_4_ 0.5, MgSO_4_ 0.3, mineral salt solution 1.0 v/v (mineral salt solution [g 50 mL^−1^]: NaCl 0.5, MnSO_4_·5 H_2_O 0.8, FeSO_4_·7 H_2_O 0.9, CuSO_4_·5 H_2_O 0.08, ZnSO_4_·7 H_2_O 0.08, CoSO_4_·7 H_2_O 0.08; pH 6.0 in purified water] with 1.5% w/v agar were prepared in advance and stored at 4°C. Serial dilution in sterile 0.9% w/v saline solution was applied to prepare the samples. The prepared dilutions were heat‐shocked at 80°C for 30 min at 500 rpm, so only germinated endospores occur as colonies on the plates after incubation. From the heat‐shocked samples 100 μL respectively were spread evenly over the prepared petri dishes using a cell spreader. Inoculated petri dishes were incubated at 37°C for 24‐48 h and the CFU was determined subsequently.

### 2.5 Ammonium iron(II) sulfate assay

The modification of the assay was based on the publications of Janssen et al. [16] and Rotman et al. [17]. After cultivating *Bacillus* spp., the samples were autoclaved for 20 min at 121°C. Subsequently a centrifugation step (15,000 × g, 15 min, 4°C) was added to release DPA from the cells and separate the DPA in the supernatant from cell debris. For the assay buffer, an acetate buffer (0.05 mol L^−1^, pH 4.6) was prepared. Additionally, a cysteine stock solution was freshly prepared (50 g L^−1^, 0.1% w/v). The composition for 1 mL of assay buffer consists of 980 μL acetate buffer and 20 μL cysteine stock solution, which is used as a blank for the detection as well. Lastly 10 mg mL^−1^ ammonium iron(II) sulfate (1% w/v) were freshly added to the assay buffer. The DPA stock solution contains 1 g L^‐1^ and can be prepared in saline solution or purified water. Using the DPA stock solution, a calibration series in the range between 0 and 200 μg mL^−1^ DPA was prepared. Samples were measured in 96‐well plates at 440 nm, each well containing 160 μL prepared sample or DPA calibration solution and 40 μL assay buffer. The calculation of the DPA concentration based on the ammonium iron(II) sulfate assay is depicted in Equation (2).

(2) $$\begin{array}{ccc} & & \mathrm{DPA}\;\mathrm{concentration}\;[\mu g\;{\mathrm{mL}}^{-1}]\\ & & \;=\frac{{\mathrm{OD}}_{440\;\mathrm{nm}}\left(\mathrm{sample}\right)-{\mathrm{OD}}_{440\;\mathrm{nm}}\left(\mathrm{blank}\right)}{\mathrm{slope}\;\mathrm{of}\;\mathrm{regression}\;\mathrm{line}} \end{array}$$

The limit of detection (LOD) was calculated as depicted in Equation (3).

(3) $$S_{\mathrm{LOD}}={\bar{S}}_{0}+3\times \sigma_{0}.$$

### 2.6 Spore detection by algorithm

For the initial endospore detection of *Bacillus* spp. and the calibration of a cell counting model, spray dried powder was used. Further experiments were conducted using *Bacillus* spp. cultivation broth. The samples were diluted if necessary and were then applied to the Petroff cell counting chamber. For image processing Aforge was used (Aforge.net (version 2.0.0, Andrew Kirillov, https://code.google.com/archive/p/aforge/downloads?page=2, date of retrieval 22.10.21). Subsequently, images of the samples were generated and transferred into the software‐based counting algorithm. The open source code can be found here: https://seafile.cloud.uni‐hannover.de/d/f0067b765c20473abed7/

## 3 RESULTS

### 3.1 Ammonium iron(II) sulfate assay

**FIGURE 1:**
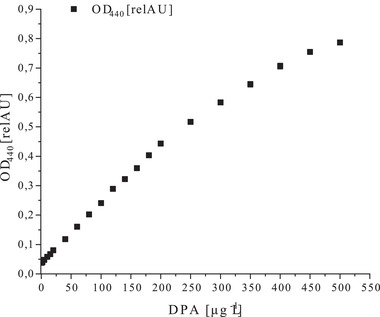
Detection of pure DPA in purified water in a concentration range between 0 and 500 μg mL^−1^ and measurement at 440 nm. A linear range between 0 and 200 μg mL^−1^ DPA is visible

Initial experiments were based on the historic publication by Janssen et al. [16] using ascorbic acid as an antioxidant in the ammonium iron(II) sulfate assay. An occurring precipitation and reddish discoloration, led to the exploration of the ammonium iron(II) sulfate assay by Rotman and Fields [17]. In this assay, cysteine was used as an antioxidant and a lower pH of the acetate buffer was set, resulting in a yellow chelate complex formed by DPA and ammonium iron(II) sulfate in purified water. First, the modification of the assay for *Bacillus* spp. was focused on the detection of pure DPA in purified water. A linear range for the detection of DPA in purified water measured at 440 nm was found between 0‐200 μg mL^‐1^ (R^2^ = 0.9997) and is depicted in Figure 1.

A screening for the optimal wavelength between 390‐550 nm for the measurement confirmed the stated wavelength of 440 nm by Rotman and Fields [17] as the optimum for this assay. Additionally, the calculated limit of detection (LOD) for pure DPA dissolved in water was LOD_H2O_ = 1.44 μg mL^‐1^ DPA. Consequently, the assay was initially tested using *B*. *coagulans* to determine the cell number by using the concentration of DPA. Therefore, it is necessary to compare established methods for the determination of cell numbers to this assay. In this work, the determination of CFU and cell counting using a Petroff counting chamber were used as established methods. The results for this can be found in Table 1. The determination of the cell count by counting chamber was set as 100% due to its reliability as an established method. The CFU determination only depicts germinated endospores and thus cannot display the total cell count. Nonetheless, CFU determination can be prone to errors as seen in the results (Table 1). Only 45% of the initial spore count by counting chamber was found using the CFU determination. By applying the ammonium iron(II) sulfate assay, *Bacillus* spp. endospores were successfully detected and the DPA content was calculated.

**TABLE 1:** Calibration of the ammonium iron(II) sulfate assay in comparison to cell count by counting chamber and CFU determination using *B. coagulans*

Method of detection	Spore number calibration [endospores mL^−1^]
Counting chamber	1.50 × 10^9^ (±1.5 × 10^8^)
CFU	6.7 × 10^8^ (±1.0 × 10^8^)
Ammonium iron(II) sulfate assay	1.53 × 10^9^ (±1.0 × 10^8^)

For the application of this assay, a cell number from 1.1 × 10^7^‐1.5 × 10^9^ endospores mL^‐1^ is advisable.

### 3.2 Spore detection by algorithm

**FIGURE 2:**
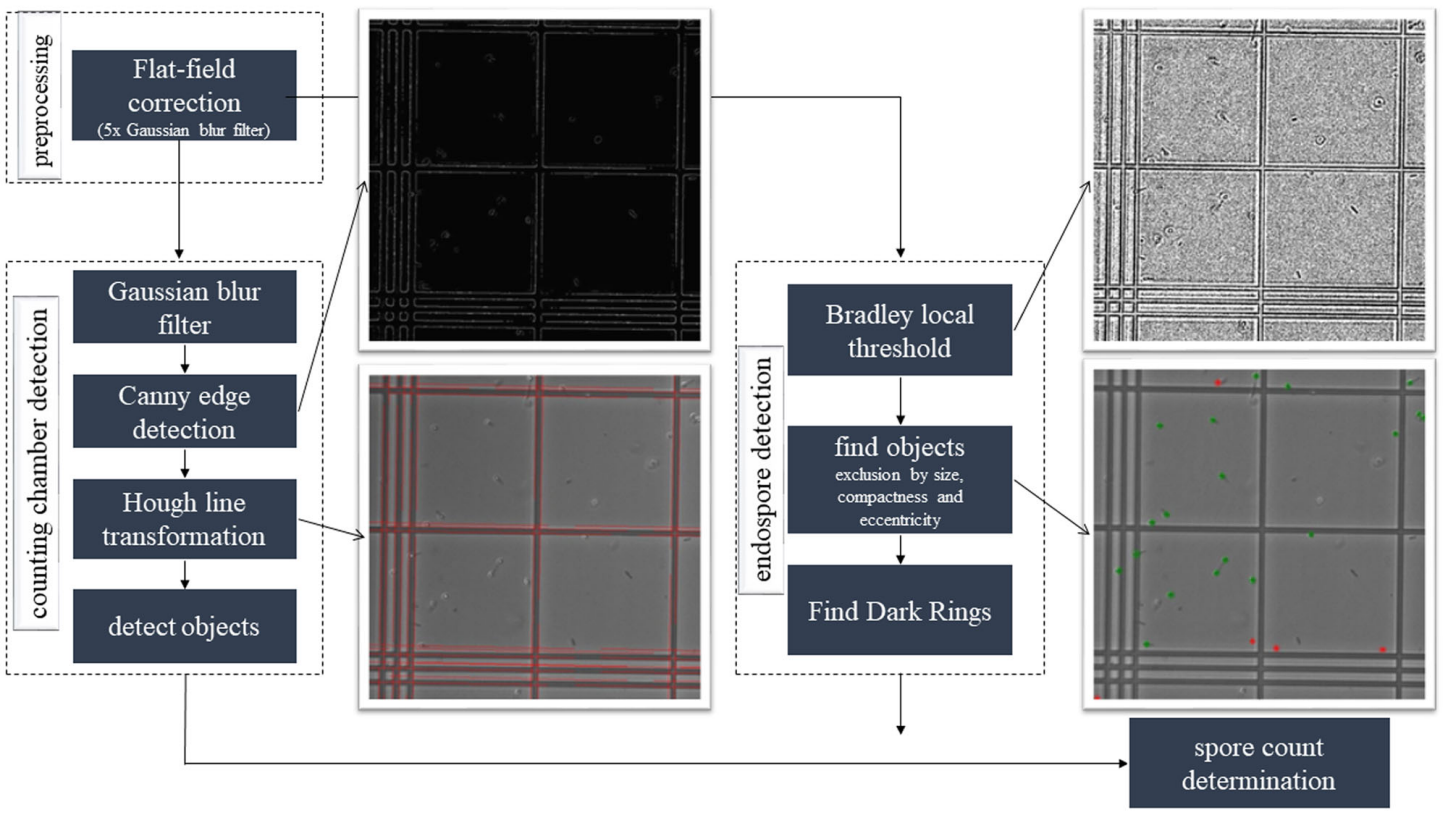
Overview of the developed counting model for the automated endospore detection of Bacillus spp

Additionally, the cell count can be determined by phase‐contrast microscopy. Phase‐contrast microscopy depicts spores as highly reflective and round structures, which can be distinguished from the vegetative cells by their shape and reflection. The development of a counting model for spore detection can simplify the detection of endospores of *Bacillus* spp. In Figure 2, an overview of the developed counting model is concluded.

First, a Flat‐field correction was applied to minimize an occurring light gradient caused by the microscope apparatus. To improve the image, a copy of the original image was created and a fivefold Gaussian blur filter with a size of 21 and a σ‐value of 5 was applied to edit the images. For the application of the Flat‐field correction, an empty image is usually used. The Gaussian blur filter was applied due to the lines of the counting chamber and resulted in the blurring of the lines to generate a nearly empty image. Subsequently, the lighting conditions were factored out by subtraction. Additionally, the Gaussian blur filter improved the edge detection in the following Canny edge detection. This detection method depicted the edges as one‐pixel‐width edges. The parameters for the detection were: lower threshold = 20, upper threshold = 41 and σ = 1.4. A detection of the edges, using Canny edge detection, is necessary to improve the following Hough line transformation. This transformation serves to find the lines in the image. For this, a fitting linear equation was generated for every white pixel. In this procedure, a Hess normal form was used to calculate the parameters radius and θ. The resulting accumulation of parameters (frequency for a calculated combination of the two parameters) was shown in a matrix, also known as a parameter space. The local maxima represent possible linear slopes [37]. Subsequently, the detected lines were sorted out over the angle and distance to each other. For spore detection, the preprocessed image was processed with a Bradley local threshold. To do so, the window size was set to 7 and the σ‐value to 0. Most endospores can be easily detected with this procedure, as a dark ring surrounds them. Then, the image was checked for objects that had a certain size. Pixels were added to an object, if they were located in its so‐called Moore neighborhood (8‐connected‐neighborhood). Since the endospores have a round shape, they were then sorted out based on their compactness and eccentricity. Due to a high amount of background noise, objects that are not endospores were also detected. Because of this, a method was inserted that makes use of the dark ring around endospores. This method, here referred to as FDR (Find Dark Rings), generates 16 lines that run out from the center of the object. Then, each pixel on a line was compared with the darkest pixel of the object. If there is a darker pixel, this concludes that the dark ring has been found correctly. In the algorithm images, this correct finding was indicated as a green line. If at least 75% of the lines of an object are marked green, it is recognized as an endospore. Finally, the detected objects were assigned to the respective counting chamber squares. The position of the detected object was compared to the straight line and was divided into a class. Each class equals a small square and was assigned a different color. The endospore number in each class was counted and displayed.

The developed model was tested by comparing its resulted spore count to a manual count in a counting chamber, a CFU determination and the application of the ammonium iron(II) sulfate assay. For these experiments three *Bacillus* strains were used, namely *B. coagulans*, *B. licheniformis*, *B. subtilis*. The results of the spore count determination by using four different methods are depicted in Table 2.

**TABLE 2:** Spore count determination of *B. coagulans*, *B. licheniformis*, and *B. subtilis* by counting chamber (manual count, set as 100%), model‐based automated cell count, ammonium iron(II) sulfate assay and CFU determination

*Bacillus* strain	Manual cell count [spores mL^‐1^] (set as reference)	Automated cell count [spores mL^‐1^]	Ammonium iron(II) sulfate assay [spores mL^‐1^]	CFU determination [CFU mL^‐1^]
*B. coagulans*	3.06 × 10^9^ (±6.66 × 10^7^)	2.80 × 10^9^ (±6.51 × 10^7^)	3.01 × 10^9^ (±1.04 × 10^8^)	2.14 × 10^8^ (±1.96 × 10^7^)
*B. licheniformis*	5.73 × 10^9^ (±1.59 × 10^8^)	4.91 × 10^9^ (±4.13 × 10^8^)	4.85 × 10^9^ (±5.07 × 10^7^)	2.02 × 10^9^ (±3.69 × 10^8^)
*B. subtilis*	1.46 × 10^8^ (±9.00 × 10^7^)	1.66 × 10^8^ (±1.19 × 10^8^)	1.31 × 10^8^ (±6.62 × 10^7^)	2.13 × 10^8^ (±1.51 × 10^7^)

The three *Bacillus* species were successfully detected by the automated cell count resulting in a correct recognition of 85.11% (±5.21%) for *B. licheniformis*, 89.23% (±3.31%) for *B. coagulans* and 86.35% (±2.90%) for *B. subtilis*. Furthermore, the ammonium iron(II) sulfate assay showed comparable results to the manually counted spore number. As expected, the CFU determination showed the largest deviation from the manual cell count by counting chamber.

**FIGURE 3:**
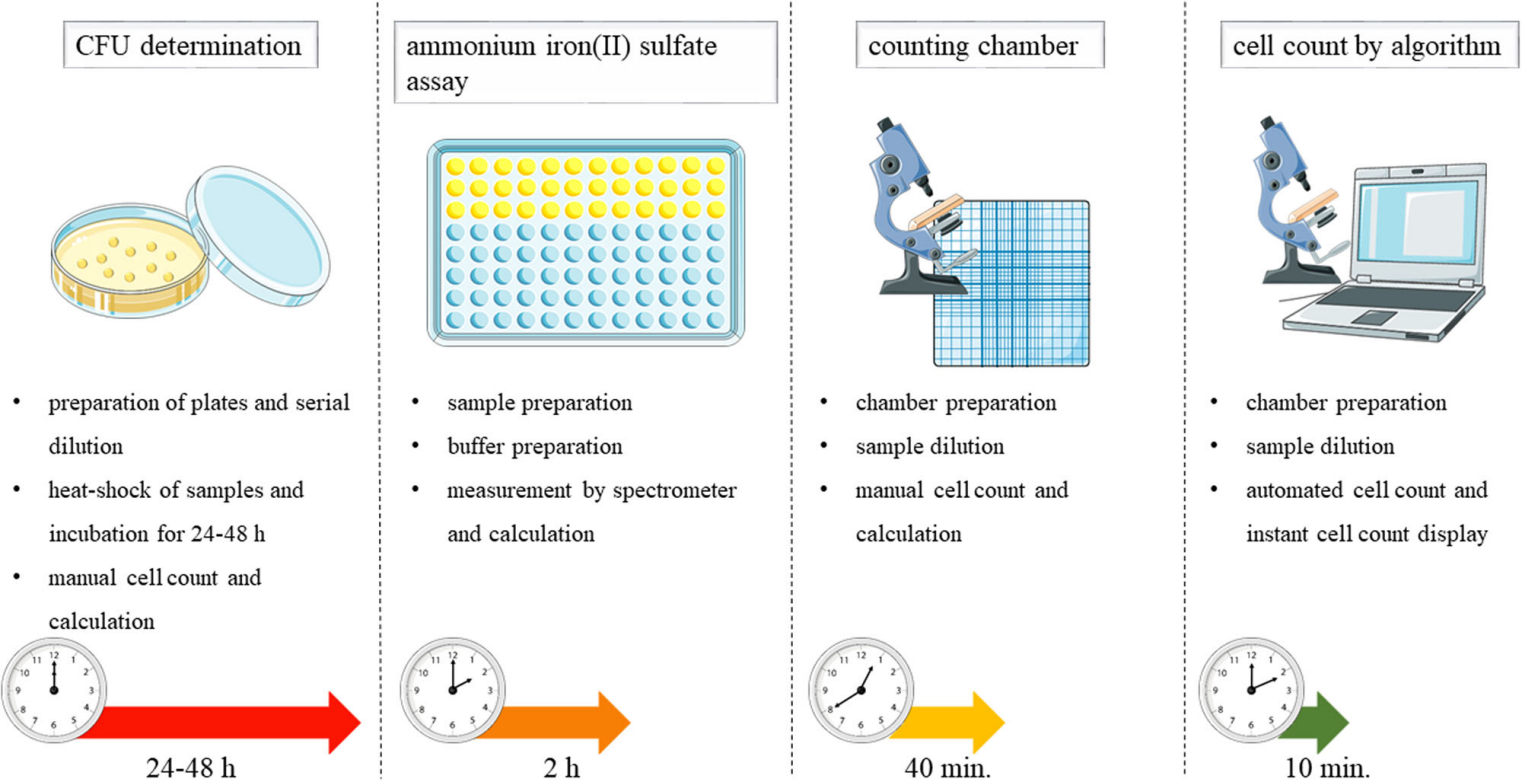
Overview of two established methods (CFU and counting chamber) and the two novel applications for the determination of Bacillus spp. endospores. Depicted are time and work consumption as well as necessary work steps for sample processing. Image requisition from smart.servier.com. Reference for images from Figure 3: Servier, “Servier Medical Art,” 2021. smart.servier.com. Date of acquisition 15.09.21

Figure 3 summarizes the estimated expenditure of time and necessary preparation steps for the four different spore count methods (CFU, ammonium iron(II) sulfate assay [16, 17], counting chamber, and cell count by algorithm), respectively.

## 4 DISCUSSION

In this work, the historical ammonium iron(II) sulfate assay by Janssen et al. and Rotman et al. [16, 17] was modified and applied to three different *Bacillus* spp. Subsequently, a linear range for the detection of DPA could be confirmed as well as the used wavelength. To establish this method in the laboratory routine, the ammonium iron(II) sulfate assay was performed and compared to CFU determination as well as the manual cell count by counting chamber. In these experiments, the ammonium iron(II) sulfate assay showed well comparable results for the endospore detection as the standard method (counting chamber). Nonetheless, this work also underlined the expenditure of time and workload as well as the immense error quota of the CFU determination. By using this method, only viable germinated endospores are detected, which results in a significantly lower and false spore count [38].

The ammonium iron(II) sulfate assay accelerates endospore detection, especially in relation to CFU determination. This method is particularly interesting for an overall screening of endospores as a contamination in various samples. However, the detection limit entails the application for food or soil samples, as endospore numbers are much lower in such samples [21, 39]. Other assay methods, such as Terbium(III) assays provide a better fit for these areas of application, but are of limited application due to the use of rare earth elements [39, 40]. A further possible source of friction can be media components, which are affecting the turbidity. These circumstances make it necessary to transfer the sample to saline.

Additionally in this work, a new algorithm for the detection of endospores in phase‐contrast microscopy was developed. Here, the basic approach utilizes the fact that bacterial endospores are highly reflecting round structures compared to the rod‐shaped vegetative cells of *Bacillus* spp. The algorithm was integrated into a pre‐existing microscopy software to automate the spore count at‐line. For this detection method, a counting chamber is used and the algorithm processes each small square defined by the edges and lines of the chamber. Per image, the algorithm needs 8 s to detect and calculate the spore count. For the application of the counting model in the laboratory routine, a comparison between the CFU, manual cell count by counting chamber and ammonium iron(II) sulfate assay were conducted for three different *Bacillus* species. The average detection percentage has been between 85‐89%. The developed counting model enables accelerated endospore counting based on one of the most reliable methods for cell counting. Therefore, this method can be established quickly and effectively into the laboratory routine. In the laboratory routine, only a counting chamber, phase‐contrast microscope and the developed software are necessary to apply this method. In addition, this method can be used to monitor and adapt an ongoing bioprocess as an at‐line measurement. Furthermore, workload and expenditure of time for plating, counting or evaluation of results are minimized. Only the set up of the counting chamber and possible dilution are necessary for pre‐processing. The application is however limited by a false‐positive spore count and can be further optimized. Motile vegetative bacteria can convey a spore‐like image as a highly reflective structure in images due to its position in the counting chamber if vertical to line of sight. These false‐positive endospores could be eliminated by generating multiple images with a certain period of time between recordings. In this case, the motile vegetative cells should not be detected over multiple timed images. Further research could be conducted in the processing of the images resulting in a total cell count (including vegetative cells). Moreover, this model can result in an automated spore and vegetative cell count and could be included at‐line or real‐time in a bioprocess. Further development of the algorithm could also lead to a deep‐learning model feeding an AI as described by Gao et al. for fungal spore detection [36].

The application of the two presented methods on environmental samples could be limited. For example, samples containing high concentrations of Calcium can potentially interfere with the colorimetric detection of DPA. In environmental samples, the developed counting model can also provide conclusive results due to the unique size and depiction of endospores in phase‐contrast microscopy. Problematic for the detection of endospores in environmental samples by the counting model could be other sample components with a high reflection and same size.

In this work, an ammonium iron(II) sulfate assay was successfully modified for the facilitated detection of bacterial endospores of *Bacillus* spp., which shortens the workload and preparation time compared to a CFU determination. Moreover, an automated counting model for the microscopical detection of endospores using phase‐contrast microscopy was developed. The application of this counting model results in a time‐saving of multiple hours up to days compared to the standardized CFU determination and improves the laboratory routine time‐ and error wise. A combination of these two methods can facilitate the endospore detection in biological samples. Possible applications can be the monitoring of spore production, e.g. in probiotics, bioprocess control or contamination monitoring.

## CONFLICT OF INTEREST

The authors declare no conflict of interest.

## DATA AVAILIBILITY STATEMENT

The data that support the findings of this study are available from the corresponding author upon request.

## DEDICATION

This article is dedicated to Prof. Dr. Thomas Scheper, Professor for Technical Chemistry and outstanding scientist with manifold research interests, who has carried out substantial biotechnological research with his exceptional expertise. With his entry into his well‐deserved retirement, the scientific community will lack from now on one of the last representatives being able to cover the whole multi‐facetted discipline of biotechnology.

## NOMENCLATURE

**Table:** 

σ	[–]	Standard deviation of the Gaussian distribution
θ	[°]	Angle of the Hess normal form

## References

[1] Higgins, D. , Dworkin, J. , Recent progress in *Bacillus subtilis* sporulation. FEMS Microbiology Reviews 2012, 36(1), 131–148. 22091839 10.1111/j.1574-6976.2011.00310.x PMC3237856

[2] Setlow, P. , Resistance of bacterial spores, in: Storz, G. , Hengge‐Aronis, R. , (Ed.), Bacterial Stress Responses, Amerian Society for Microbiology, Washington D.C 2000, pp. 217–230.

[3] Setlow, P. , Minireview: I will survive: Protecting and repairing spore DNA. J. Bacteriol. 1992, 174(9), 2737‐2741. 1569005 10.1128/jb.174.9.2737-2741.1992 PMC205922

[4] Bressuire‐Isoard, C. , Broussolle, V. , Carlin, F. , Sporulation environment influences spore properties in Bacillus: Evidence and insights on underlying molecular and physiological mechanisms. FEMS Microbiology Reviews 2018, 42(5), 614–626. 29788151 10.1093/femsre/fuy021

[5] Murrell, W. G. , Chemical composition of spores and spore structures. Bact. Spore 1969, 7, 215–73.

[6] Errington, J. , *Bacillus subtilis* sporulation: Regulation of gene expression and control of morphogenesist. Microbol Rev., 1993, 57(1), 1‐33. 10.1128/mr.57.1.1-33.1993 PMC372899 8464402

[7] Andreoli, A. J. , Saranto, J. , Baecker, P. A. , Suehiro, S. , et al. Biochemical properties of forespores isolated from *Bacillus cereus* , in: Gerhardt, P. , Costilow, N. , Sadoff, H. L. , (Ed.), Spores VI, American Society for Microbiology, Washington D.C. 1975, 418‐424.

[8] Fritsche, O. , Überlebensstrukturen, in: Fritsche, O. , (Ed.), Mikrobiologie, Springer Berlin, Heidelberg 2016, pp. 29.

[9] Setlow, P. , Germination of spores of Bacillus species: What we know and do not know. J. Bacteriol. 2014, 196(7), 1297–1305. 24488313 10.1128/JB.01455-13 PMC3993344

[10] Elisashvili, V. , Kachlishvili, E. , Chikindas, M. L. , Recent advances in the physiology of spore formation for Bacillus probiotic production. Probiotics Antimicrob. Proteins 2019, 11(3), 731–747. 30515722 10.1007/s12602-018-9492-x

[11] Sutton, S. , The limitations of CFU: compliance to CGMP requires good science. J. GXP Compl. 2012, 16(1), 74–80.

[12] Gorsuch, J. P. , Jones, Z. , Le Saint, D. , Kitts, C. L. , Enumeration of industrial Bacillus assemblages in commercial products with customized plate‐counting assays. J. Microbiol. Methods 2019, 164, 105682. 31381982 10.1016/j.mimet.2019.105682

[13] Davis, C. , Enumeration of probiotic strains: Review of culture‐dependent and alternative techniques to quantify viable bacteria. J. Microbiol. Methods 2014, 103, 9–17. 24814752 10.1016/j.mimet.2014.04.012

[14] Schaeffer, A. B. , Fulton, M. D. , A simplified method of staining endospores. Science 1933, 77(1990), 194. 17741261 10.1126/science.77.1990.194

[15] Hamouda, T. , Shih, A. Y. , Baker, J. R. , A rapid staining technique for the detection of the initiation of germination of bacterial spores. Lett. Appl. Microbiol. 2002, 34(2), 86–90. 11849500 10.1046/j.1472-765x.2002.01047.x

[16] Janssen, F. , Lund, A. , Anderson, L. , Colorimetric assay for dipicolinic acid in bacterial spores evidence for a new growth‐promoting acid produced by *Lactobacillus casei* . Science 1957, 127(5), 5–6. 10.1126/science.127.3288.26 13495474

[17] Rotman, Y. , Fields, M. L. , A modified reagent for dipicolinic acid analysis. Anal. Biochem. 1968, 22(1), 168. 5636949 10.1016/0003-2697(68)90272-8

[18] Koo, T. M. , Ko, M. J. , Park, B. C. , Kim M. S. , et al. Fluorescent detection of dipicolinic acid as a biomarker in bacterial spores employing terbium ion‐coordinated magnetite nanoparticles. J. Hazard. Mater. 2021, 408, 124870. 33387720 10.1016/j.jhazmat.2020.124870

[19] Alp, M. , Pamuk Algi, M. , Algi, F. , Eu(III)–DO3A and BODIPY dyad as a chemosensor for anthrax biomarker. Luminescence 2021, 1–8. 10.1002/bio.4129 34337847

[20] Xiu, L. F. , Huang, K.Y. , Zhu, C. T. , Zhang, Q. , et al. Rare‐earth Eu3+/gold nanocluster ensemble‐based fluorescent photoinduced electron transfer sensor for biomarker dipicolinic acid detection. Langmuir 2021, 37(2), 949–956. 33405936 10.1021/acs.langmuir.0c03341

[21] Hindle, A. A. , Hall, E. A. H. , Dipicolinic acid (DPA) assay revisited and appraised for spore detection. Analyst 1999, 124(11), 1599–1604. 10746319 10.1039/a906846e

[22] Navarro, A. K. , Peña, A. , Pérez‐Guevara, F. , Endospore dipicolinic acid detection during *Bacillus thuringiensis* culture. Lett. Appl. Microbiol. 2008, 46(2), 166–170. 18069985 10.1111/j.1472-765X.2007.02277.x

[23] Kong, L. , Zhang, P. , Wang, G. , Yu, J. , et al. Characterization of bacterial spore germination using phase‐contrast and fluorescence microscopy, Raman spectroscopy and optical tweezers. Nat. Protoc. 2011, 6(5), 625–639. 21527920 10.1038/nprot.2011.307

[24] He, L. , Liu, Y. , Lin, M. , Mustapha A. , et al. Detecting single Bacillus spores by surface enhanced Raman spectroscopy. Sens. Instrum. Food Qual. Saf. 2008, 2(4), 247–253.

[25] Farquharson, S. , Gift, A. D. , Maksymiuk, P. , Inscore, F. E. , Rapid dipicolinic acid extraction from Bacillus spores detected by surface‐enhanced Raman spectroscopy. Appl. Spectrosc. 2004, 58(3), 351–354. 15035719 10.1366/000370204322886735

[26] Scully, M. O. , Kattawar, G. W. , Lucht, R. P. , Opatrny, T. , et al. FAST CARS: Engineering a laser spectroscopic technique for rapid identification of bacterial spores. Proc. Natl. Acad. Sci. U. S. A. 2002, 99(17), 10994–11001. 12177405 10.1073/pnas.172290899 PMC123198

[27] Majeed, M. , Majeed, S. , Nagabhushanam, K. , Punnapuzha, A. , et al. Rapid assessment of viable but nonculturable *Bacillus coagulans* MTCC 5856 in commercial formulations using flow cytometry. PLoS One 2018, 13(2), 1–14. 10.1371/journal.pone.0192836 PMC5825061 29474436

[28] Tabor, M. W. , MacGee, J. , Holland, J. W. , Rapid determination of dipicolinic acid in the spores of Clostridium species by gas liquid chromatography. Appl. Environ. Microbiol. 1976, 31(1), 25–28. 942206 10.1128/aem.31.1.25-28.1976 PMC169712

[29] Mawatari, K. , Atsumi, M. , Nakamura, F. , et al. Determination of dipicolinic acid in ‘Natto’ by high‐performance liquid chromatography coupled with postcolumn photoirradiation with zinc acetate. Int. J. Tryptophan Res. 2019, 12(2), 2–6. 10.1177/1178646919852120 PMC6585242 31258330

[30] Kong, L. , Doona, C. J. , Setlow, P. , Li, Y. Q. , Monitoring rates and heterogeneity of high‐pressure germination of bacillus spores by phase‐contrast microscopy of individual spores. Appl. Environ. Microbiol. 2014, 80(1), 345–353. 24162576 10.1128/AEM.03043-13 PMC3911021

[31] Wagner, J. , MacHer, J. , Automated spore measurements using microscopy, image analysis, and peak recognition of near‐monodisperse aerosols. Aerosol Sci. Technol. 2012, 46(8), 862–873.

[32] Li, J. , Huang, J. , Shen, L. , Ye, Q. , et al. A Method of Human Interaction based semi‐automatic Counting for Digital Microscope Image of Spore. Chinese Automation Congress, 2020, 3217–3221.

[33] Harrold, Z. R. , Hertel, M. R. , Gorman‐Lewis, D. , Optimizing *Bacillus subtilis* spore isolation and quantifying spore harvest purity. J. Microbiol. Methods 2011, 87(3), 325–329. 21989299 10.1016/j.mimet.2011.09.014

[34] Lei, Y. , Yao, Z. , He, D. , Automatic detection and counting of urediniospores of *Puccinia striiformis* f. sp. tritici using spore traps and image processing. Sci. Rep. 2018, 8(1), 1–11. 30206343 10.1038/s41598-018-31899-0 PMC6134082

[35] Korsnes, R. , Westrum, K. , Fløistad, E. , Klingen, I. , Computer‐assisted image processing to detect spores from the fungus *Pandora neoaphidis* . MethodsX 2016, 3, 231–241. 27073786 10.1016/j.mex.2016.03.011 PMC4810014

[36] Gao, W. , Li, M. , Wu, R. , Du, W. , et al. The design and application of an automated microscope developed based on deep learning for fungal detection in dermatology. Mycoses 2021, 64(3), 245–251. 33174310 10.1111/myc.13209

[37] Steinbrecher, R. Bildverarbeitung in der Praxis, R. Oldenbourg, München 1993, pp. 233–241.

[38] Turnbull, P. C. B. , Frawley, D. A. , Bull, R. L. , Heat activation/shock temperatures for *Bacillus anthracis* spores and the issue of spore plate counts versus true numbers of spores. J. Microbiol. Methods 2007, 68(2), 353–357. 17055602 10.1016/j.mimet.2006.09.014

[39] Fichtel, J. , Köster, J. , Scholz‐Böttcher, B. , Sass , H., et al. A highly sensitive HPLC method for determination of nanomolar concentrations of dipicolinic acid, a characteristic constituent of bacterial endospores. J. Microbiol. Methods 2007, 70(2), 319–327. 17573136 10.1016/j.mimet.2007.05.008

[40] Fichtel, J. , Köster, J. , Rullkötter, J. , Sass, H. , Spore dipicolinic acid contents used for estimating the number of endospores in sediments. FEMS Microbiol. Ecol. 2007, 61(3), 522–532. 17623026 10.1111/j.1574-6941.2007.00354.x

